# Atypical presentation of a late-onset blue nevus

**DOI:** 10.1016/j.jdcr.2022.10.014

**Published:** 2022-10-27

**Authors:** Monik Gupta, Rohun Gupta, Preeti Jhorar

**Affiliations:** a The University of Toledo Health Science Campus, Toledo, Ohio; b Oakland University William Beaumont School of Medicine, Rochester, Michigan; c Department of Dermatology, Kaiser Permamenete, Downey, California

**Keywords:** atypical nevus, melanoma, psoriasis

## Introduction

Blue nevi are melanocytic proliferations that are reminiscent of embryonal neural crest–derived melanocytic precursors.^1^ Normally, melanocytes migrate to the epidermis from the neural crest; however, blue nevi are thought to occur because of premature arrest of melanocyte migration.^1^

Blue nevi most commonly arise in children and young adults as either congenital or acquired lesions. In addition, blue nevi have a general 2:1 predilection for women than for men. There are multiple variants of blue nevi, and these include common, cellular, amelanotic, combined, sclerosing or desmoplastic, epithelioid, and subungual. The 2 most common subtypes are common and cellular blue nevi.^1^

Common blue nevi are flat or dome shaped with a smooth surface and are typically 0.5 to 1 cm in diameter.^2^ On the contrary, cellular blue nevi are characterized by a nodular appearance and are 1 to 3 cm in diameter.^2^ Although common blue nevi are found on the dorsal surface of the hands and feet, cellular blue nevi are located in the gluteal and sacrococcygeal regions.^3^ In addition, blue nevi may be found in the head and foot regions.^4^ Common blue nevi are often diagnosed in younger adults, whereas cellular blue nevi are typically seen in middle-aged individuals.^3^^,^^5^

The purpose of this case report is to present a unique presentation of a large, benign, late-onset, blue nevus.

## Case report

**Fig 1:**
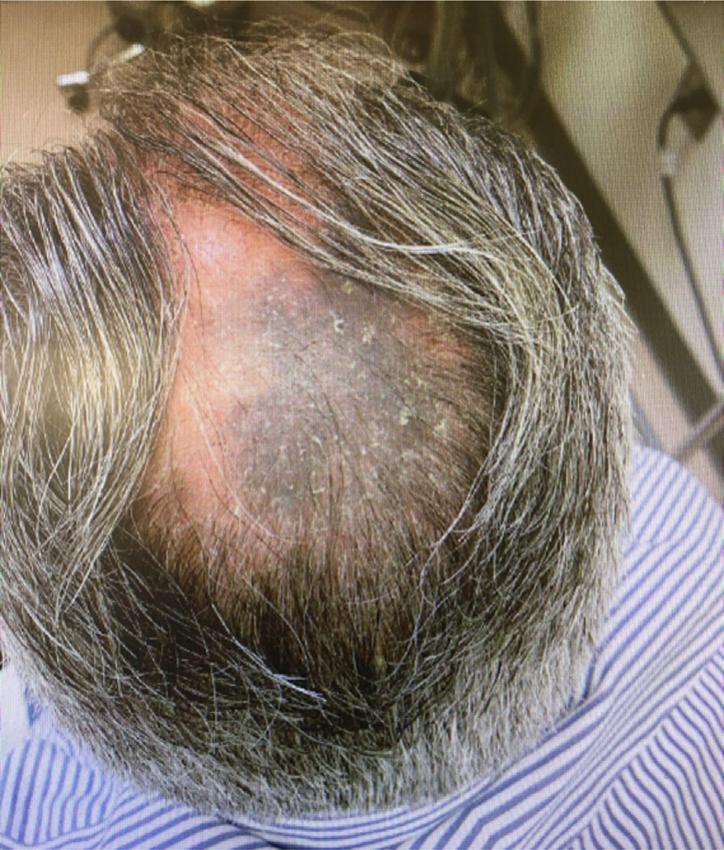
Large, homogeneous, blue-gray plaque with whitish scales on the scalp, with overlying whitish, silvery scales.

A 54-year-old man with a significant history of psoriasis presented at his follow-up appointment, and upon physical examination, a 12 × 7-cm^2^ bluish, homogenous patch with overlying whitish scales was visualized on the apex of his scalp (Fig 1). Upon questioning, he believed that the patch emerged approximately 5 to 6 years ago, which was confirmed by his partner. Because of uncertain onset and the large size of the nevus.

A punch biopsy was performed, which revealed dendritic melanocytes to be associated with a collagenous stroma and melanophages in the upper dermis. Spongiotic changes overlay these findings. Based on these results, we categorized the lesion as an unusually large common blue nevus. Given the large size of the nevus, the patient was offered excision; however, the patient declined to undergo the procedure. Instead, he agreed to follow-up for further monitoring.

**Fig 2:**
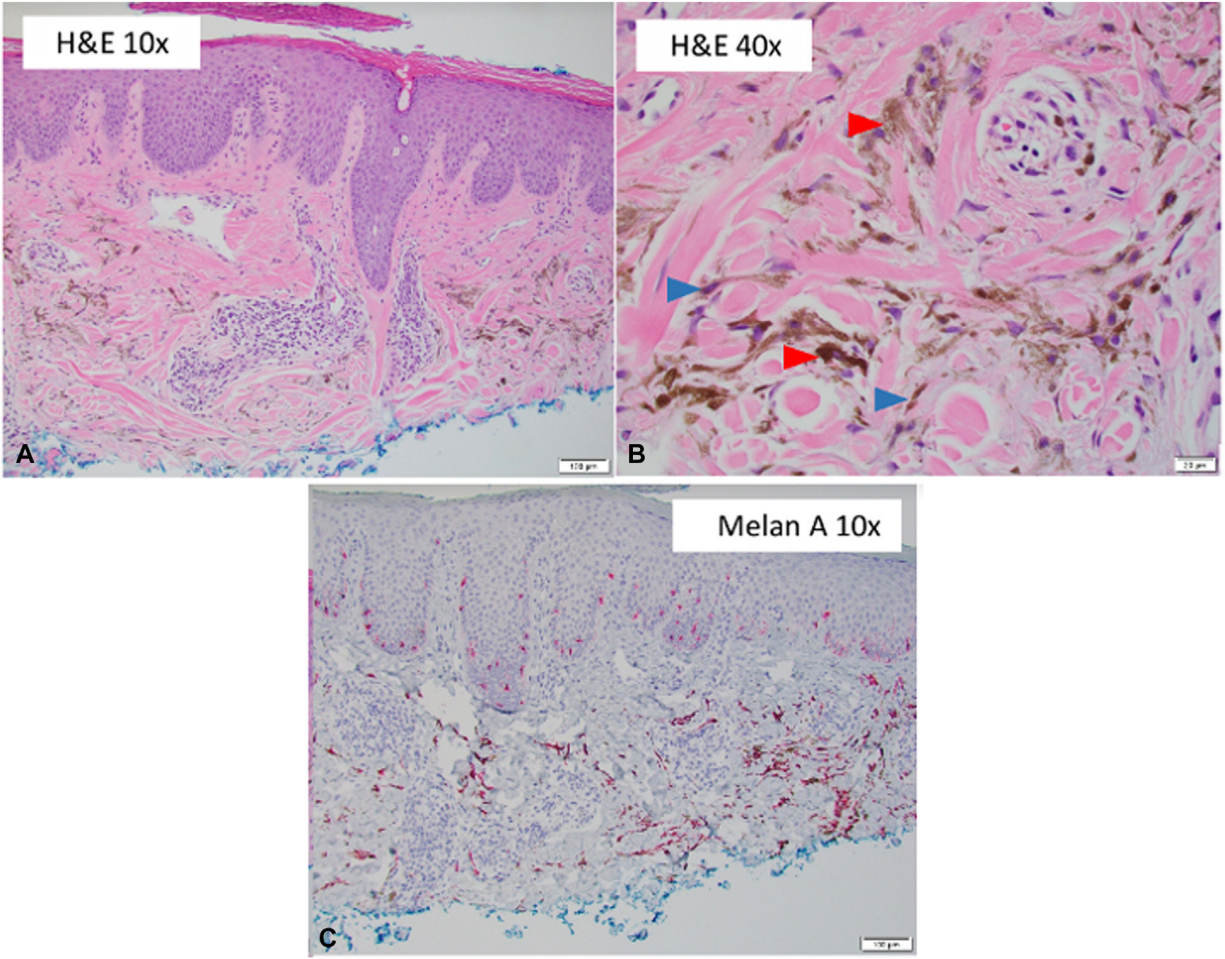
Nevus pathology. **A,** A low-power view of blue nevus showed 2 foci of pigmented cells in a collagenous stroma in the superficial dermis, suggesting a benign blue nevus (hematoxylin and eosin stain; original magnification: ×10.) **B,** A high-power view showed 2 cell types: spindled-to-dendritic melanocytes (*blue arrow*) and melanophages (*red arrow*) (hematoxylin and eosin stain; original magnification: ×40.) **C,** A low-power view showed melanocytes highlighted by immunostaining for melan A (melan A stain; original magnification: ×10.)

Three scouting biopsies were performed to sample different areas of the nevus at a follow-up appointment. The biopsy report, showing a benign blue nevus with other changes (Fig 2, *A-C*), revealed folliculitis, with no signs of architectural changes that would indicate malignancy. The patient continues to follow-up every 4 to 6 months and has agreed to additional biopsies for monitoring of the nevus.

## Discussion

Blue nevi are clinically diagnosed using dermatoscopes and have been described to have a steel blue color that gradually fades into the surrounding skin.^6^ They have characteristic homogenous yet structureless pigmentation.^6^ With regard to genetics, the true mechanism of blue nevi is uncertain. However, recent studies have suggested that an acquired *BRAF*^*V600E*^ mutation is the culprit behind the proliferation and premature arrest in melanocytes.^7^ Furthermore, ∼80% of blue nevi present with genetic mutations in the *GNAQ* gene, and ∼15% of blue nevi present with mutations in the *GNA11* gene.^8^^,^^9^ Malignant blue nevi and blue nevus-like melanomas tend to have additional mutations in the *SF3B1* and *BAP1* genes.^9^

Biopsy and pathologic evaluation are the gold standards to rule out malignancy.^3^ When a provider encounters such a large blue nevus, he or she must be prudent to perform a biopsy of any suspicious areas of the lesion that are seen with or without a dermatoscope. If the lesion appears homogenous, then it is appropriate to perform a biopsy of various locations to get a better understanding of the nevus.

Blue nevi range in size, and malignancy may be suspected if a lesion is found to be larger than 6 mm in diameter. Although there are no definitive size guidelines available regarding blue nevi, the literature often applies the same parameters that govern conventional nevi to blue nevi. Studies have suggested that nevi >20 cm are at a 5% to 60% increased risk of becoming malignant. Medium-sized nevi that range between 1.5 to 19.9 cm have a <5% increased risk of becoming malignant. Additional observational studies are important for enhancing our understanding of the relationship between the size of blue nevi and the risk associated with their transformation into malignant melanomas.^10^

Typically, a benign nevus that is acquired throughout the lifetime of a patient requires no treatment other than longitudinal monitoring. It is critical to note that acquired nevi may increase the risk of melanoma, and, as a result, patients should be counseled on sun protection and undergo full-body skin examinations. Although small and stable blue nevi, often ranging from 0.5 to 3 cm, require no clinical intervention, late-onset atypical lesions should be biopsied because of reports of melanoma arising from cellular blue nevi.

## Conflicts of interest

None disclosed.
